# *Nigella sativa*-based agar medium for identifying *Cryptococcus neoformans*

**DOI:** 10.22034/cmm.2026.345553.1772

**Published:** 2026-02-01

**Authors:** Hossein Khodadadi, Faezeh Rouhi, Ghasem Mohammadi Nematabad, Erfan Khodadadi

**Affiliations:** 1 Department of Medical Parasitology and Mycology, School of Medicine, Shiraz University of Medical Sciences, Shiraz, Iran; 2 Basic Sciences in Infectious Diseases Research Center, Shiraz University of Medical Sciences, Shiraz, Iran; 3 Faculty of Medicine, Najafabad Branch, Islamic Azad University, Najafabad, Isfahan, Iran

**Keywords:** Black seed, *Cryptococcus neoformans*, *Nigella sativa*, Selective media

## Abstract

**Background and Purpose:**

*Cryptococcus neoformans* is an important opportunistic fungus that causes severe infections, including cryptococcal meningitis, especially in immunocompromised patients. One of its key characteristics is the ability to produce melanin from melanin precursors. This pigmentation serves as a useful marker to distinguish fungus colonies from other yeasts when grown on selective media, such as bird seed agar (BSA). However, the complicated preparation of medium and high cost of commercial BSA restrict its routine application. This study aimed to present and assess a new culture medium prepared from black seed (*Nigella sativa*) meal extract (NSME) as an affordable and more accessible alternative to BSA for the identification of *C. neoformans*.

**Materials and Methods:**

Various NSME-based media formulations, with and without *N. sativa* oil (NSO), were prepared and tested against type strains, clinical and environmental isolates of *Cryptococcus* species, and other medically relevant yeasts. Growth rate and melanin-associated pigmentation of the yeast colonies were assessed during 14 days of incubation.

**Results:**

The NSME media effectively promoted the growth and melanin production of *C. neoformans* with colony pigmentation ranging from light to dark brown depending on NSME concentration. In contrast, NSO had minimal impact on growth or pigmentation. Addition of 1% glucose to newly developed media enhanced growth rates, aligning colony sizes with those observed on Sabouraud dextrose agar. Non-melanin producer yeasts, such as *Candida* spp. and *Saccharomyces cerevisiae*, showed limited growth and no pigmentation, facilitating species differentiation.

**Conclusion:**

The NSME-based culture media offer a reliable, low-cost alternative for the growth and preliminary identification of *C. neoformans*, with promising potential for use in routine diagnostics, especially in low-resource settings.

## Introduction

*Cryptococcus neoformans* and *Cryptococcus gattii* are opportunistic basidiomycetous yeasts responsible for cryptococcosis, a life-threatening infection primarily affecting the lungs and central nervous system. Annually, hundreds of thousands of cryptococcal meningitis cases occur worldwide, with estimated mortality rates ranging from 41% to 61%. While the *Cryptococcus* genus includes multiple species, *C. neoformans* accounts for the majority of clinical cases [1]. According to the World Health Organization Fungal Priority Pathogens List, *C. neoformans* is classified as a top-ranking pathogen within the critical priority group [2].

Despite advancements in laboratory methods for the detection and identification of this yeast, culture-based isolation remains a fundamental step in its diagnosis. Other conventional diagnostic approaches for cryptococcosis include histopathology, direct microscopy, fungal culture, and cryptococcal antigen detection [3].

A key characteristic of *C. neoformans* is its ability to produce melanin pigment through phenoloxidase activity, which catalyzes the oxidation of various O-phenolic compounds, such as L-dopa, L-dopamine, chlorogenic acid, and caffeic acid. Synthesis of melanin represents a key phenotypic characteristic that differentiates *C. neoformans* from other similar yeasts within the genus and plays a significant role in its pathogenicity. The melanin production in culture media supplemented with melanin precursors leads to the formation of dark-pigmented yeast colonies, facilitating the differentiation of this species from other yeasts [4-6].

Bird seed agar (BSA), also known as Niger Seed Agar or Staib Agar, which contains *Guizotia abyssinica* seed extract, is widely used for the selective isolation and differentiation of *C. neoformans*. On this medium, *C. neoformans* produces characteristic light-to-dark-brown colonies due to melanin synthesis. Various culture media enriched with melanin precursors have been evaluated for their ability to detect melanin-producing yeasts. These media include formulations supplemented with bird seed, caffeic acid, henna, mustard seed, tobacco, sunflower seed extracts, and other plant-based substrates [7-11], with variable effectiveness. Nevertheless, there is a continuing demand for alternative culture media that are cost-effective, widely accessible, and efficient in facilitating melanin production for the identification of *C. neoformans*. However, the high cost and limited availability of some of those substrates restrict their widespread use, highlighting the necessity of finding an alternative compound to make new diagnostic media [12].

*Nigella sativa* is an annual flowering plant that belongs to the family Ranunculaceae. Its seeds are commonly known as black cumin or black seed. This herbaceous plant is native to Southern Europe, North Africa, and parts of Asia, including India and the Middle East. Its seeds have been used for centuries in traditional medicine and cooking practices. The seeds contain bioactive compounds, such as thymoquinone, dihydrothymoquinone, dithymoquinone, and thymol, as well as essential oils and unsaturated fatty acids, like linoleic and oleic acid. In addition, they are a rich source of essential amino acids, vitamins, and minerals. Notably, *N. sativa* seeds encompass a variety of melanin precursors, making them a promising candidate for making fungal differential culture media [13]. These include tyrosine, an essential amino acid directly involved in melanin biosynthesis, and phenylalanine, which acts as a precursor to tyrosine and further contributes to melanin production. Additionally, thymoquinone and dihydrothymoquinone, two key constituents of *N. sativa*, have been shown to modulate melanin synthesis by affecting tyrosinase activity [14].

Given the extensive use of BSA for the identification of *C. neoformans* and the growing demand for alternative culture media, this study aimed to develop and assess a novel *N.sativa*-based agar (NSA) for the rapid and precise detection of *C. neoformans*. By evaluation of its melanin production efficiency in comparison to traditional BSA, this research aimed to establish a new, reliable medium for the identification of *C. neoformans* from both clinical and environmental samples.

## Materials and Methods

### Preparation of media containing Nigella sativa extract

Various NSA media were formulated using a water extract of whole *N. sativa* seed meal, whole oil seeds, and combinations of different ratios of oil-free meal extract and *N. sativa* oil (NSO). The seeds were obtained from an herbal market in Shiraz, Iran, and authenticated by a botanist. Their purity was verified through visual inspection to ensure the absence of contamination with other seeds. To extract the oil, the seeds were processed using a cold-press extraction system (ODK 40, Isfahan Osveh Sanat Company, Isfahan, Iran). The extracted oil was collected in sealed glass containers, and the remaining oil-free seed meal was separated and stored for subsequent use. The *N. sativa* meal extract (NSME) was prepared by drying the meal in a hot-air oven at 70 °C, followed by fine grinding with an electric grinder. To produce the extract, 400 g of the powdered seed meal was mixed with 1,000 mL of distilled water at room temperature and then boiled for 20 min. After cooling to room temperature, the mixture was filtered twice through multiple layers of sterilized gauze to obtain the final extract. This extract was used to prepare growth media with varying concentrations of meal extract, including 1%, 5%, 10%, and 15% per liter. Additionally, media were formulated to evaluate the effects of *N. sativa* oil, both individually and in combination with the extract. Since the oil extracted from the seed is approximately half of its total weight, an equal proportion combination was used.

After adding granulated agar (Merck KGaA, Darmstadt, Germany) and dissolving the components, the media were autoclaved at 121 °C for 15 min, and the pH was adjusted to 5.6 as needed. The specific composition and quantities of each medium are detailed in Table 1.

**Table 1:** Ingredients of different *Nigella sativa*-based culture media, incorporating *N. sativa* meal extract (NSME), *N. sativa* oil (NSO), and their combination, were prepared in a final volume of one liter using distilled deionized water.

Culture media	*Nigella sativa* Extract	*Nigella sativa* Oil	Glucose	Agar
1% NSME	10 ml	-	-	15 g
5% NSME	50 ml	-	-	15 g
10% NSME	100 ml	-	-	15 g
15% NSME	150 ml	-	-	15 g
1% NSME + 1% NSO	10 ml	10 ml	-	15 g
5% NSME + 5% NSO	50 ml	50 ml	-	15 g
10% NSME + 10% NSO	100 ml	100 ml	-	15 g
15% NSME + 15% NSO	150 ml	150 ml	-	15 g
NSO 10%	-	100 ml	-	15 g
10% NSME + 1% Glucose	100 ml	-	10 g	15 g

### Preparation of bird seed agar

To prepare the BSA medium, 50 g of *Guizotia abyssinica* seed extract was dissolved in 500 mL of distilled water, following the same method used for the NSA medium. The extract was then combined with 1 g of glucose, 15 g of agar (Merck), and additional distilled water to reach a final volume of one liter. The mixture was heated until all components were fully dissolved and the solution became uniform. The medium was then autoclaved as previously described, and the pH was adjusted to 5.6 as needed.

### Preparation of Sabouraud dextrose agar

Commercial Sabouraud dextrose agar (SDA, Merck KGaA, Darmstadt, Germany) was prepared according to the instructions of the manufacturer. This medium was utilized for the general cultivation of fungi and preparation of yeast suspensions from freshly grown colonies.

All media were supplemented with 20 units/mL of penicillin and streptomycin (Norbrook, UK) to inhibit bacterial growth.

### Yeast and type-strains, inoculation, and growth conditions

A diverse set of type strains, along with clinical and environmental isolates of *Cryptococcus* species and other clinically important yeast species, was used to assess the performance of a newly developed NSA medium in comparison with conventional and BSA media. The tested yeasts included *Cryptococcus neoformans*, *Cryptococcus gattii*, *Cryptococcus albidus* (*Naganishia albida*), *Candida albicans*, *Candida krusei* (*Pichia kudriavzevii*), *Candida glabrata* (*Nakaseomyces glabratus*), *Candida tropicalis*, *Candida parapsilosis*, and *Saccharomyces cerevisiae* (Table 2).

**Table 2:** Yeast and type strains used for the evaluation of media.

	Yeast strain	Reference No.
1	*Candida albicans*	ATCC 10231
2	*Candida parapsilosis*	ATCC 22019
3	*Candida tropicalis*	TIMM 0313
4	*Candida glabrata* (*Nakaseomyces glabratus*)	ATCC 90030
5	Candida krusei (Pichia kudriavzevii )	ATCC 6258
6	*Cryptococcus albidus* (*Naganishia albida*)	Environmental isolate*
7	*Cryptococcus gattii*	ATCC 14248
8	*Cryptococcus neoformans*	Environmental isolate*
9	*Cryptococcus neoformans*	ATCC 62066
10	*Cryptococcus neoformans*	Clinical isolate†
11	*Saccharomyces cerevisiae*	PTCC 5177

* These strains were recovered from environmental samples in Shiraz, Iran, and identified through polymerase chain reaction sequencing of rDNA region.

† The yeast was isolated from a confirmed case of Cryptococcal meningitis and identified using the same sequencing method.

Fresh colonies of each yeast species were added to a Falcon tube containing 10 mL of sterile distilled water (deuterium-depleted water) to prepare uniform suspensions with a concentration of 1–2 × 10^6^ CFU/mL, equivalent to the 0.5 McFarland standard.

A 10 μl droplet of each yeast suspension was spot-plated at the center of the media plates and incubated at 30 °C for two weeks. Colony growth rate (GR) and pigmentation were monitored at 3-, 7-, and 14-day intervals to assess the effects of the different media formulations. The extended period up to 14 days ensures sensitivity for slow-growing or low-level isolates and follows standard clinical mycology protocols for a definitive negative result. The experiments were conducted in duplicate and repeated to ensure the reliability and reproducibility of the results.

### Colony growth rate and colony color assessment

The GRs were analyzed descriptively to highlight biologically relevant patterns across media and incubation periods. This method allowed identification of differential growth behaviors and provided insights into the effects of media composition and incubation time on microbial development. Similar approaches were applied and discussed in previous studies [15, 16].

In this study, the following grading scale was established and applied to assess colony growth based on the diameter of colonies on days 3, 7, and 14. Colony growth was categorized as follows: 3-5 mm (1+), 5-8 mm (2+), 8-10 mm (3+), and >10 mm (4+), with no growth considered negative (-).

In addition, fungal growth and morphological characteristics, such as changes in colony color (CC) and texture on the new media, were compared with those observed on standard BSA and SDA media. The GR and CCs of various yeast species were then assessed across media containing different concentrations of NSMEs, with a particular emphasis on evaluating the potential of these media for the isolation and differentiation of *C. neoformans*. Changes in CC on the NSAs were documented and compared to those on SDA and BSA.

### Statistical analysis

The GR was recorded semi-quantitatively using an ordinal scale (1+ to 4+). Since each species/medium/incubation time combination had a single observation, inferential statistical testing with independent replicates was not applicable. Therefore, the collected data were analyzed descriptively.

## Results

The GR increased with incubation time across most culture media for all tested yeast species (Supplementary Table 1). Enriched media, including SDA and NSME supplemented with oil or glucose, consistently supported higher GR scores (3+ to 4+) at days 7 and 14. In contrast, basal NSME and BSA media exhibited limited growth, particularly at early incubation times. These growth patterns were consistently observed across Candida spp., Cryptococcus spp., and S. cerevisiae, indicating a time-dependent and medium-dependent growth response. Key growth patterns observed at the optimal incubation time of 7 days are summarized in Table 3.

**Table 3:** Growth rate (GR) and colony characteristics of isolates across *Nigella sativa* oil (NSO), Nigella sativa meal extract (NSME), Sabouraud dextrose agar (SDA), and bird seed agar (BSA) culture media at 7 days of incubation.

	Media	1% NSME	5% NSME	10% NSME	15% NSME	1% NSME + 1% NSO	5% NSME + 5% NSO	10% NSME + 10% NSO	15% NSME + 15% NSO	10% NSO	10% NSME +1% Glucose	BSA	SDA
*Candida albicans*	GR	2+	2+	2+	2+	2+	2+	2+	2+	1+	3+	2+	3+
	CC	W	W	W	W	W	W	W	W	W	W	W	W
*Candida parapsilosis*	GR	2+	2+	2+	2+	2+	2+	2+	2+	1+	3+	3+	3+
	CC	W	W	W	W	W	W	W	W	W	W	W	W
*Candida tropicalis*	GR	2+	2+	2+	2+	2+	2+	2+	2+	2+	2+	3+	3+
	CC	W	W	W	W	W	W	W	W	W	W	W	W
*Cryptococcus gattii*	GR	1+	1+	2+	2+	1+	1+	2+	2+	1+	2+	2+	2+
	CC	LB	LB	DB	DB	LB	LB	DB	DB	LB	DB	DB	W
*Cryptococcus neoformans* *	GR	1+	1+	2+	2+	1+	2+	2+	2+	1+	2+	2+	3+
	CC	LB	LB	DB	DB	LB	LB	DB	DB	LB	DB	DB	W
*C. neoformans* †	GR	2+	2+	2+	2+	2+	2+	2+	2+	2+	2+	3+	3+
	CC	DB	DB	DB	DB	DB	DB	DB	DB	LB	DB	DB	W
*C. neoformans* †	GR	1+	1+	2+	2+	1+	1+	2+	2+	0	2+	2+	2+
	CC	LB	LB	LB	LB	LB	LB	LB	LB	-	DB	DB	W
*Cryptococcus albidus*	GR	1+	2+	2+	2+	1+	1+	2+	2+	0	2+	2+	2+
	CC	W	W	W	W	W	W	W	W	-	W	W	W
*Candida glabrata*	GR	2+	2+	2+	2+	2+	2+	2+	2+	1+	2+	2+	3+
	CC	W	W	W	W	W	W	W	W	W	W	W	W
*Candida krusei*	GR	2+	2+	2+	2+	2+	2+	2+	2+	0	2+	2+	3+
	CC	W	W	W	W	W	W	W	W	-	W	W	W
*Saccharomyces cerevisiae*	GR	1+	1+	2+	2+	1+	1+	2+	2+	0	2+	2+	3+
	CC	W	W	W	W	W	W	W	W	-	W	W	W

GR grading was based on colony diameter: 3–5 mm (1+), 5–8 mm (2+), 8–10 mm (3+), >10 mm (4+), and no growth (-). CC: Colony color, W: white, LB: light brown, DB: dark brown.

* A type strain of Cryptococcus neoformans (ATCC 62066),

† A clinical isolate of Cryptococcus neoformans.

The GR grades and CC of the cultivated yeast species, following incubation for 3, 7, and 14 days on media containing NSO, *N. sativa* extract, and on control media (SDA and BSA), were recorded and are presented in the Supplementary Table. All newly developed NSA media containing NSME supported yeast growth and facilitated melanin production by *C. neoformans* at various levels. Notably, species such as *C. gattii* and *C. neoformans* exhibited enhanced growth and more obvious colony pigmentation, especially when supplemented with higher concentrations of 10-15% NSME. These media formulations facilitated robust growth, with *C. neoformans* showing a significant increase in GR by day 7, particularly on media containing glucose, which appeared to favor the growth of the species (Figure 1).

**Figure 1:**
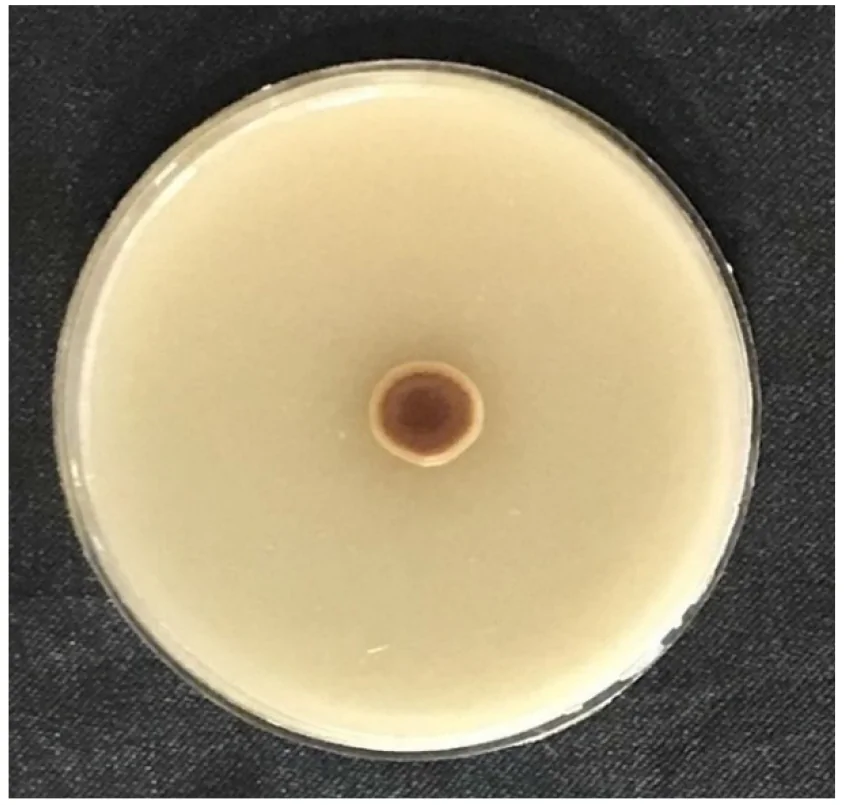
*Cryptococcus neoformans* colony characteristics on *Nigella sativa* meal extract-based medium (10% *N. sativa* meal extract) after 7 days of incubation at 30 °C.

Additionally, darker CCs were observed, transitioning from light brown to dark brown from day 3 to day 7 on media with enriched NSME concentrations (Supplementary Figure S1 and S2). *Cryptococcus neoformans* produced progressively darker colony pigmentation, transitioning from light brown to dark brown during the late first week of incubation on media enriched with higher concentrations of NSME.

*N. sativa*–based media demonstrated a distinct color change accompanied by rapid growth of *C. neoformans*, comparable to that observed on BSA. Nevertheless, *C. albidus*, *S. cerevisiae*, and *Candida* species demonstrated slower growth and maintained white colonies, making them easily distinguishable from *C. neoformans*, which could be critical in a diagnostic context.

In contrast to the rich medium SDA, BSA and the NSA did not support the growth of larger yeast colonies during the initial days of incubation. However, after an extended incubation period of 7-14 days, the colony diameters were similar in size to those on SDA. After 3, 7, and 14 days of incubation on media containing only NSO, limited yeast growth and faint colony coloration were observed across various *Cryptococcus* species colonies. Furthermore, increasing the amount of NSO did not lead to a significant improvement in the performance of the NSME-containing media.

The observed results suggest that the GR and color change of *Cryptococcus* strains were primarily affected by the quantity of meal extract rather than the oil content of black seed.

## Discussion

The present study evaluated the impact of NSME and NSO on the growth and colony pigmentation of various yeast species, with a particular focus on *C. neoformans*. The findings highlighted the differential effects of these media components on yeast growth dynamics and melanin production, offering insights into their potential diagnostic utility.

All tested NSA media supplemented with NSME supported yeast growth and allowed for observable melanin production, particularly in *C. neoformans* and *C. gattii*. These findings are consistent with the known metabolic capacity of *C. neoformans* to synthesize melanin via laccase-mediated oxidation of phenolic compounds, which are likely more abundant in the meal extract than in the oil. The pigmentation (from light brown to dark brown) observed by day 7 on NSME-enriched media reinforces this association and suggests that NSME provides essential precursors or cofactors for colony melanization. Therefore, the intensity of pigmentation of *C. neoformans* colonies on NSME media could serve as a practical phenotypic marker to distinguish *C. neoformans* from non-melanizing yeasts, such as *Candida* spp., *C. albidus*, and *S. cerevisiae*, which remained non-pigmented throughout the incubation period.

While the melanogenesis-stimulating activity of *N. sativa* extract has been demonstrated in eukaryotic systems, including mammalian-derived cells, evidence in fungi is indirect [17-19]. In melanized fungi, such as *Cryptococcus* spp., melanin production is substrate-dependent and relies on the availability of exogenous phenolic or aromatic compounds oxidized by fungal laccase.

Usefulness of the NSME for the identification of *Cryptococcus* isolates can be attributed to the presence of melanogenic precursors within the extract, including tyrosine, phenolic, and quinone compounds, such as thymoquinone [20]. These constituents provide suitable substrates for oxidative polymerization into melanin-like pigments, resulting in visible brown to black pigmentation comparable to that observed on conventional melanin-inducing media. Collectively, the NSME creates a favorable environment for fungal melanization, enhancing pigment expression and supporting the differentiation and presumptive identification of *Cryptococcus* and other melanin-producing fungi in clinical mycology.

Growth-promoting effect of glucose, particularly when supplemented in NSME media, was another noteworthy observation. Inclusion of 1% glucose significantly enhanced the GR of *C. neoformans*, which was especially evident on days 7 and 14, aligning colony sizes with those observed on rich media, like SDA. This suggests that while NSME supplies melanin precursors and other nutrients, it may be suboptimal in terms of carbon source availability, a limitation that can be mitigated through glucose supplementation.

Culture media prepared solely with NSO showed minimal effectiveness in promoting the GR or CC development of *Cryptococcus* yeasts compared with media containing meal extract alone or in combination with oil. This limited performance may be explained by several factors. The seed meal appears to contain higher concentrations of essential nutrients and substrates required for yeast growth and melanin production, including sugars, proteins, and polyphenolic compounds, which are largely absent from the extracted oil. In contrast, the hydrophobic nature of NSO may lead to the formation of a superficial oil layer on the culture medium, potentially acting as a physical barrier that restricts nutrient diffusion and limits yeast access to underlying substrates.

These characteristics likely explain the weak colony development and minimal pigmentation observed on NSO-only media across all incubation periods. Additionally, the standard formulations of SDA and BSA contain small amounts of glucose, resulting in slightly higher GRs compared with NSME. However, this difference could be effectively compensated for by supplementing NSME with 1% glucose, supporting the role of readily available carbohydrates in enhancing yeast growth.

The delayed but eventual similarity in colony size between SDA and NSME media after 14 days of incubation is also noteworthy. While initial growth on SDA was more robust, likely due to its nutrient-rich formulation, prolonged incubation allowed NSME-based media to support comparable colony expansion. This delayed growth progress emphasizes the need for extended incubation when using selective or differential media, such as NSME-based formulations.

From a diagnostic perspective, NSME-based media offer a dual advantage. They not only facilitate the selective growth of *C. neoformans* but also induce the development of characteristic colony pigmentation, which may serve as a readily observable diagnostic indicator. In clinical contexts, particularly in resource-limited settings, such media can support the identification of *Cryptococcus* species while minimizing reliance on more complex biochemical or molecular assays. Moreover, the use of these media aligns with the strategic objectives of the United Nations Sustainable Development Goals by fostering circular bioeconomy principles that promote the utilization of renewable resources. This enhances ecological resilience through the employment of safe and natural byproducts in place of complex synthetic compounds.

In summary, the findings suggest that NSME is a more effective component than its oil counterpart for developing culture media aimed at promoting the growth and melanization of *C. neoformans*. The collected data support the use of NSME-based formulations, particularly when supplemented with glucose, as practical and low-cost tools for differentiating melanin-producing yeast *C. neoformans* from other yeasts in diagnostic mycology.

Future studies should explore the optimization of NSME concentrations, examine the biochemical constituents responsible for melanin synthesis, and assess the utility of these media in clinical sample screening.

## Conclusion

This study demonstrated that NSME-based media provide a cost-effective and accessible alternative to traditional BSA for the cultivation and preliminary identification of *Cryptococcus neoformans*. The NSME-supported media not only facilitate yeast growth but also promote melanin production, resulting in a distinct colony pigmentation pattern that can aid in differentiating *C. neoformans* from non-melanizing yeasts. Compared to BSA, which depends on commercially prepared *Guizotia abyssinica* seed extract and may be costly or difficult to obtain in some regions, NSME is derived from a widely available and inexpensive natural product. This makes NSME-based formulations particularly suitable for laboratories in resource-limited settings.
